# Speciation in Metal Toxicity and Metal-Based Therapeutics

**DOI:** 10.3390/toxics3020170

**Published:** 2015-04-28

**Authors:** Douglas M. Templeton

**Affiliations:** Department of Laboratory Medicine and Pathobiology, University of Toronto, 1 King’s College Circle, Toronto, ON M5S 1A8, Canada; E-Mail: doug.templeton@utoronto.ca; Tel.: +1-416-978-3972; Fax: +1-416-978-5959

**Keywords:** metal speciation, therapeutic metallocomplexes, metal chelation, nickel, platinum, gold, iron

## Abstract

Metallic elements, ions and compounds produce varying degrees of toxicity in organisms with which they come into contact. Metal speciation is critical to understanding these adverse effects; the adjectives “heavy” and “toxic” are not helpful in describing the biological properties of individual elements, but detailed chemical structures are. As a broad generalization, the metallic form of an element is inert, and the ionic salts are the species that show more significant bioavailability. Yet the salts and other chelates of a metal ion can give rise to quite different toxicities, as exemplified by a range of carcinogenic potential for various nickel species. Another important distinction comes when a metallic element is organified, increasing its lipophilicity and hence its ability to penetrate the blood brain barrier, as is seen, for example, with organic mercury and tin species. Some metallic elements, such as gold and platinum, are themselves useful therapeutic agents in some forms, while other species of the same element can be toxic, thus focusing attention on species interconversions in evaluating metal-based drugs. The therapeutic use of metal-chelating agents introduces new species of the target metal *in vivo*, and this can affect not only its desired detoxification, but also introduce a potential for further mechanisms of toxicity. Examples of therapeutic iron chelator species are discussed in this context, as well as the more recent aspects of development of chelation therapy for uranium exposure.

## 1. Introduction

The concept of speciation is fundamental to understanding metal toxicity, and before we can understand the therapeutic approaches available for metal poisoning, it is necessary to appreciate that how a metal damages the organism, and how it can safely be removed from the body, are exercises in speciation. Our approach in this review will be to introduce the importance of speciation with a particularly rich example, that of nickel, and then present some definitions and concepts related to metal speciation and metal toxicity, with brief examples of how different structural levels of speciation come into play in metal toxicity. We will then look at some examples of metal poisoning resulting from species-dependent use of metals as therapeutic agents, and finally will examine chelation therapy of metal intoxication as a process that must incorporate principles of speciation, using iron and uranium as examples. We will refer to each metallic element by its chemical symbol M alone when no species information is indicated; use M^0^ to denote the pure elemental form, refer to oxidation states in compounds or generic reactions as M(nI), and use superscripts M^n+^ to indicate a specific available ionic species. For example, we will use to Ni in referring generically to nickel, Ni^0^ to denote elemental nickel metal, Ni(II) to specify the oxidation state in a compound such as Ni(II)subsulfide (Ni_3_S_2_), or Ni^2+^ when referring to the exchangeable aqueous divalent ion.

## 2. Importance of Speciation: The Example of Ni

Nickel serves as a good example of the critical importance of speciation in toxicology, and of how this is sometimes surprisingly overlooked. The International Agency for Research on Cancer has concluded that there is sufficient evidence that Ni compounds are carcinogenic to humans (classified by IARC as Group 1), with the strongest human data relating to inhalation of aerosols of Ni(II) salts and less soluble oxidic and sulfidic species, whereas Ni^0^ is possibly carcinogenic (IARC Group 2B) [1]. However, among Ni compounds, a wide range of carcinogenic potential is observed (summarized here from [2]). Soluble Ni(II) salts are of low acute toxicity upon oral exposure, and indeed the soluble Ni^2+^ ion is found in a number of foods and beverages. Thus, accidental ingestion of up to 2.5 g Ni^2+^ as NiCl_2_ and NiSO_4_ in drinking water produced only mild and transient effects [3]. Solid evidence that ingestion of these salts leads to cancer is lacking. On the other hand, in experimental animals, α-trinickel disulfide [Ni(II)subsulfide, Ni_3_S_2_] is a potent carcinogen. An order has been established of carcinogenicity in rats as Ni_3_S_2_ ~ β-nickel(II)sulfide (NiS) > nickel(II)oxide (NiO) >> Ni^0^ >>> amorphous NiS, for nickel particles injected intramuscularly. Comparison of the carcinogen Ni_3_S_2_ with the relatively non-carcinogenic amorphous NiS illustrates the importance of phagocytosis in getting higher quantities of Ni(II) into the cell (and nucleus) from crystalline Ni_3_S_2_. The carcinogenic effects of nuclear Ni(II) appear to be epigenetic; a recent study has characterized a number of Ni(II)-binding sites in histones; [4] that may affect gene expression. In a cell culture assay, Ni_3_S_2_, NiS, and NiO showed morphological evidence of transformation, whereas NiCl_2_ and NiSO_4_ did not, and only NiO caused neoplastic transformation [5]. Nickel sulfides and oxides are quite insoluble in water, but may become biologically available upon interaction with biological ligands. Occupational exposures to Ni usually involve multiple species. For instance, workers may be exposed to Ni_3_S_2_, NiSO_4_, NiCl_2_, NiO, NiCO_3_, Ni^0^, Ni-Fe oxides, and Ni-Cu oxides in various smelting and refining operations [1,2], and sorting out bioactive species in a given scenario becomes difficult. Inorganic ligands also affect particle size and surface chemistry of Ni, and this in turn contributes to properties such as protein adsorption and bioavailability.

Despite the wealth of information on Ni speciation, then, it is rather surprising that one still often encounters the blanket statement that “nickel is carcinogenic”. In an early study of human toxicokinetics, the author ingested 20 µg Ni/kg body wt. as an aqueous solution of Ni(NO_3_)_2_ enriched to 88% in the stable isotope ^61^Ni [6]. This represents about 1.2 mg of Ni^2+^. For comparison, this amount of Ni may typically be found in about 150 g of dried tea leaves that leaches as aqueous Ni^2+^ during infusion, and is less that ten times the average daily dietary intake of total Ni in North American and European diets. However, citing Ni as a carcinogen, permission was not given by a Canadian research ethics board to extend the study to other volunteers. Subsequently, Danish authorities, taking speciation and route of administration into account, did allow a similar protocol in Danish volunteers in a stable isotope study designed to look at toxicokinetic parameters in individuals with Ni allergy [7].

## 3. Definitions and Terminology: Speciation and Some Imprecise Terms

### 3.1. Definition Related to Speciation

According to the International Union of Pure and Applied Chemistry (IUPAC) [8], a chemical species is the “specific form of an element defined as to isotopic composition, electronic or oxidation state, and/or complex or molecular structure”. “Speciation” can be defined as the distribution of an element among defined chemical species in a system, and “speciation analysis” as the analytical activities of identifying and/or measuring the quantities of one or more individual chemical species in a sample. The structural levels at which speciation is defined (that is, isotopic composition, electronic or oxidation state, and/or complex or molecular structure) are somewhat arbitrary, but imply the requirement for a certain level of kinetic and thermodynamic stability to allow both structural designation and analytical measurement. For example, a metal ion bound to two isoforms of a protein with defined amino acid sequences could be considered to be present as two distinct species, but an ion bound to a polyelectrolyte such as humic acid or heparin would not be defined in terms of multiple species representing individual molecules in the heterogeneous and polydisperse population. In this case, it is advisable to refer to fraction. “Fractionation” has been defined as the process of classification of an analyte or group of analytes from a certain sample according to physical (e.g., size, solubility) or chemical (e.g., bonding, reactivity) properties [8]. Physicochemical properties and dimensions of particulates are not considered in the IUPAC definition of speciation, which is restricted to defined molecular structures as considered in more detail in the following section. Thus, particulates are not considered in this review. However, it should be noted that properties such as hydrodynamic or aerodynamic size, surface area, and surface charge will all affect the way particulates interact with biological systems and bioaccessibility of chemical species that may be released from them. Furthermore, the increasing medical and industrial use of nanoparticles, and recognition of their prevalence in our environment, is beyond a discussion of speciation, but defines a new branch of toxicology [9].

### 3.2. Structural Considerations

According to the above definitions, elemental speciation must consider various levels of structure, some of which are more relevant than others to bioavailability, biodistribution, and toxicity. This topic has been reviewed elsewhere [2,10,11], and a few examples, briefly described, will suffice here.

The isotopic abundances of an element can differ between samples for several different reasons. If one or more isotopes arises from radioactive decay of another element, isotopic abundances may differ with geological source. For example, ^204^Pb is of primordial origin, ^206^Pb and ^207^Pb arise as decay products of U, and ^208^Pb is a product of Th decay. Because the ratios of Pb, Th, and U will differ between different geological formations, the Pb isotope ratios can provide an isotopic signature of the metal that may help to identify sources of exposure. Separation of isotopes may also occur due to physical forces or microbial action in the environment. Differences in isotopic mass can lead to both chemical and inertial separation; oxygen may partition between two phases where it is bound in different O species, giving rise to differential enrichment of ^16^O and ^18^O. Disproportionation of S is produced by S-metabolizing bacteria that results in a different ^34^S content of sulfates and sulfides. These examples of O and S can be useful in environmental studies. Differences in isotopic composition also arise from anthropogenic activity, as seen in the variable ^6^Li/^7^Li ratios that occur because of enrichment of ^6^Li for use as a moderator in nuclear reactors. In general, however, differences in isotopic composition have minimal impact on elemental toxicity.

On the other hand, the oxidation state of different species of an element is often one of the most important features determining its toxicity, affecting its absorption, membrane transport, and excretion, as well as its toxicity at the cellular or molecular target. Examples of elements with more than one biologically important oxidation state are given in Table 1. There is no general means of predicting how the oxidation state of a particular element will affect toxicity. A Parkinson-like syndrome (manganism) has been associated with environmental occupational exposure to Mn(II), and susceptibility may be conferred by mutation in a gene Park9 that encodes a P-type ATPase functioning as a putative Mn(II) transporter. Binding of Cu(II) and Mn(II) to peptides derived from the Park9 protein has recently been reported [12]. However, inorganic Mn(III) species are generally more toxic than other oxidation states of the element; Mn(II)Cl_2_ and Mn(IV)O_2_ are both less toxic *in vitro* than Mn(III)pyrophosphate. Likewise, Cr(III) is probably an essential element, but Cr(VI) is genotoxic and carcinogenic, and Cr(VI) is better absorbed than Cr(III) both in the gut and through the skin. Chromium (VI) is taken up by some cells as CrO_4_^2−^ via anion transporters, whereas Cr^3+^ cations cross lipid membranes poorly. In contrast to Cr and Mn, where the higher oxidation states are more toxic, more reduced species of inorganic As are more toxic than higher oxidation states, in general following the order AsH_3_ > arsenites [As(III)] > arsenates [As(V)]. While oxidation state does not appear to be very important in determining As bioavailability, phosphate transporters in renal epithelia and anion exchangers in erythrocytes transport As(V) species as a phosphate mimic. The same is true of V species, and V(V); is more toxic than V(IV).

The inorganic ligands of an element can have profound effects on its bioavailability and toxicity, affecting properties such as charge, solubility, and diffusion coefficient, and so determine absorption and transport in an organism. A good example is Ni, discussed above. Hydrolysis of metal ions [M^n+^ + nH_2_O → M(OH)_n_↓ +nH^+^] is an important feature of their chemistry, and formation of metal hydroxides in aerobic aqueous solution at neutral pH is often a key determinant of their solubility and bioavailability. While the pH of the blood plasma, and the interstitial and intracellular fluids are generally well maintained close to 7.4, the content of the stomach is typically at pH 1.5–3, returning towards pH 7 during passage through the duodenum. So, it can be expected that release of many metals as the soluble M^n+^ ion will transiently increase during passage through the stomach. Another location where metal ions many theoretically be liberated is in the lysosomes, where acidification to pH 4.5–5 occurs. Such a mechanism is also involved in release of Fe^3+^ from transferrin in the endosome following receptor-mediated endocytosis.

Complexation with organic acids is another aspect of speciation that can greatly increase absorption. For instance, administration of citrate with Al(OH)_3_ rapidly increases serum Al levels in human volunteers, and Al is readily available from a diet supplemented with Al(III) lactate. Maltol enhances the gastrointestinal absorption of Al(III) and allows it to cross the blood-brain barrier. Small organic molecules affect cellular uptake in generally unpredictable ways. For instance, binding of Cd(II) to albumin renders it unavailable for uptake by cells, but removal of Cd(II) from albumin by small organic molecules facilitates its uptake by the liver. On the other hand, the bioavailability of Ni(II) is decreased by some organic ligands, such as histidine and cysteine; Stability constants of metal-ligand complexes are an important feature in predicting biological properties.

When a metallic element forms a bond with carbon that has strong covalent character, as distinct from organic chelates of metal ions, the resulting organometallic species acquires new biological properties. Such species may arise in the environment (e.g., as occurs in the environmental alkylation of Hg by microorganisms to form CH_3_Hg^+^), or may be of anthropogenic origin (e.g., the manufacture of tetraalkyl Pb compounds), or they may occur within the body itself during metabolism (e.g., the production of mono- and dimethyl As species during the metabolism of inorganic As). Methylation of metals generally increases their toxicity by making them more lipid soluble and facilitating their transport across lipid barriers such as the cell membrane or blood-tissue barriers (e.g., the blood-brain barrier, blood-testis barrier, and blood-placenta barrier). This is true of methyl derivatives of Ge, Hg, Pb, Sb, and Sn, and the brain, the testes, and the fetus are particularly susceptible to organometallic species of these elements. On the other hand, metabolic methylation of; the semi-metals As and Se aids in their detoxification.

**Table 1:** Some biologically important oxidation states of elements.

Symbol	Atomic number	Name	Oxidation states
V	23	Vanadium	IV/V
Cr	24	Chromium	III/VI
Mn	25	Manganese	II/III/IV
Fe	26	Iron	0/II/III
Co	27	Cobalt	II/III
Ni	28	Nickel	II/IV
Cu	29	Copper	0/I/II
Zn	30	Zinc	0/II
As	33	Arsenic	III/V
Se	34	Selenium	II/IV/VI
Mo	42	Molybdenum	II/III/IV/VI
Pd	46	Palladium	II/IV
Ag	47	Silver	0/I/II
Sn	50	Tin	II/IV
Sb	51	Antimony	III/V
Te	52	Tellurium	0/II/IV/VI
Pt	78	Platinum	II/IV
Au	79	Gold	0/I/III
Hg	80	Mercury	0/I/II
Tl	81	Thallium	I/III
Pb	82	Lead	II/IV
U	92	Uranium	III/VI
Pu	94	Plutonium	III/IV/V/VI

The macromolecular level of speciation is structurally the least defined, although some authors refer to the "metallome". Practically, complexation of a metal with a given protein of unique amino acid sequence and a globally averaged tertiary structure should be considered a single species, even though a sample of the metalloprotein will contain an ensemble of molecules in different states of protonation and local conformations. A detailed discussion of metalloproteins, then, can be considered relevant to speciation is beyond the scope of this review, but some general comments can be added. Many enzymes exploit metal ions for structural stability. Others impart thermodynamic effects on catalysis (e.g., conformational allostery or induction of strain at the active center (the entatic state [13]). Acid-base catalysis, and (or) redox properties are also central to many enzyme catalytic mechanisms. In some instances, the same element can serve multiple roles in the same enzyme. An example is the occurrence of two Zn^2+^ ions in alcohol dehydrogenase, one of which stabilizes protein structure while the other serves as a Lewis acid polarizing the substrate oxygen atom. Another important aspect of metal-protein interactions is the selective passage of ions such as Na^+^, K^+^ or Ca^2+^ through protein-based ion channels, to achieve signaling. In addition to passive conductance down a concentration gradient, energy-dependent protein transporters pump ions against gradients. Examples of interest in the context of human Menkes and Wislon diseases, respectively, are the copper transporters ATP7A and ATP7B. Interaction of copper with thiol groups in the 'tail' of the proteins delivers the ion to an ATP-dependent transport domain for export from the cell or delivery to intracellular organelles. And a channel that conducts down a concentration gradient can nevertheless be energy-dependent, an example being the cystic fibrosis transmembrane conductance regulator (CFTR), which uses ATP hydrolysis to regulate Cl^-^ channel opening in lung epithelium. An interesting discussion of which elemental properties suit them for which types of functions, for instance, acid-base or redox catalysis, structural stabilization, or signaling, was given by R.J.P. Williams a number of years ago [13].

Sequestration of elements by essential metalloproteins is also an important determinant of toxicity and of our understanding of toxicological mechanisms. Some proteins dominate the speciation of particular elements, as do for example transferrin with plasma Fe and metallothionein with cytosolic Cd. This achieves the safe transport of Fenton-active Fe^3+^ in the circulation, for example, or, in the case of metallothionein, sequestration of Cd^2+^ or excess Cu^+/2+^ in the liver. Diferric transferrin, Cd/Cu/Zn-metallothionein, and the copper protein ceruloplasmin are often used as standards in speciation analyses of biological fluids.

### 3.3. Imprecise Terms

Two additional terms, “heavy metal” and “toxic metal”, are in common use, but are not in keeping with the principles and goals of the precise chemical terminology that is necessary to attribute biological effects to specific entities. In both cases, the adjective can simply be dropped without loss of meaning.

Duffus [14] has pointed out the difficulties with the term “heavy metal, and has documented at least 38 different attempted definitions of the term that have been offered in the literature, including those based on density of the pure element (above 4, above 5, above 6, above 7, …), based on atomic mass (greater than Na, greater than 40, “high”, “relatively high”, …), based on atomic number (greater than 20, higher than Ca, between 21 and 92, “in the rectangular block of the Periodic Table”, …), or based solely on toxicity (such as “used in industry and generally toxic to animals”). Another problem is that the term is often used to included elements, presumably based on their toxic properties, that are not metals at all, but semi-metals such as As and Se. Clearly the breadth of definitions arises because the term serves no needed purpose, and IUPAC considers it to be an “Erroneous term used commonly in the toxicological literature but having no generally agreed meaning, sometimes even applied to nonmetals, and therefore a source of confusion and to be avoided” [15].

To understand the problem with “toxic metal”, one need only consider the widely paraphrased dictum of Paracelsus, that everything is a poison and it is only a matter of the dose. Manganese is a required cofactor in a number of enzymes of metabolism and, importantly, in the antioxidant enzyme superoxide dismutase. It thus has a recommended daily intake and is considered an essential element in human health. Nickel, on the other hand, is probably not essential in higher animals, and is often referred to as a “toxic metal”. Comparing data from the U.S. Public Health Service Agency for Toxic Substances and Disease Registry (ASTDR), MnCl_2_ has an LD_50_ by gavage in rats of 350–400 mg/kg, while the corresponding value for NiCl_2_ is 50–150 mg/kg. However, the oral LD_50_ values in rats for less-soluble NiO and Ni_3_S_2_ are > 3.6 g Ni/kg; while LD_50_ values for 1–21 days of exposure to MnCl_2_ range from 0.2 to 1 g Mn/kg/day. Species of both elements are toxic at air levels encountered in industry. Of course, toxicity depends on elemental species, dose, and route of administration, and the adjective “toxic” to describe a metal without further qualification is not meaningful.

## 4. Speciation-Based Metal Therapy: Au and Pt

Armed with a knowledge of the role speciation plays in the interactions of metals with biological systems, we can begin to exploit more systematically the therapeutic potential of metals and semi-metals. Historically, use of the toxic properties of arsenicals and mercurials were used to treat a number of disorders, notably syphilis, exploiting their toxic effects on microorganisms and often with severe adverse consequences. Here, the development of species-specific drugs in modern medicine is described briefly using as examples two metals, Au and Pt, that among other properties share the ability to cause immunosensitization.

### 4.1. Gold Compounds

**Figure 1:**
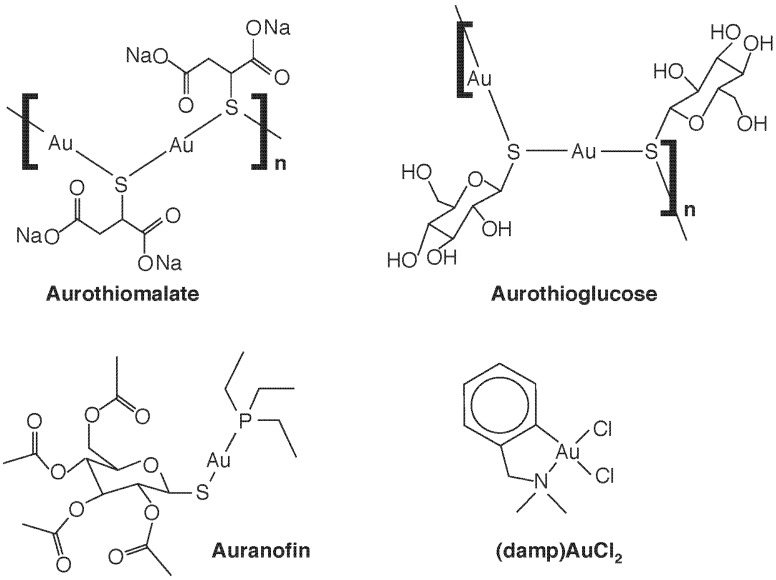
Some therapeutic gold complexes. The Au(I) species sodium aurothiomalate, aurothioglucose, and auranofin, and the Au(III) species 2-[(dimethylamino)methyl]phenyl Au(III)Cl_2_, (damp)AuCl_2_, are shown.

Gold compounds have been used therapeutically for some time—Na[Au(III)Cl_4_] joined Hg and As compounds as a treatment for syphilis in the 19th C—but it was Robert Koch who noted the bacteriostatic effects of a Au(I) compound, K[Au(CN)_2_], against *Mycobacterium tuberculosis*, and thus gold cyanide was introduced as a treatment for tuberculosis in the 1920’s [16,17]. This was ineffective and was abandoned after the advent of streptomycin, but the incorrect notion that the tubercle bacillus caused rheumatoid arthritis lead to its administration to arthritis patients. Thus, by the 1960’s, some efficacy of the use of gold compounds against rheumatoid arthritis was noted, and gold therapy or “chrysotherapy” with “gold salts” gained acceptance. The most-used compounds were not salts at all, but organic compounds containing Au(I), such as auranofin, aurothioglucose and aurothiomalate (Figure 1). Development of these drugs, and their extension to use in other forms or arthritis, such as psoriatic arthritis, juvenile arthritis, and arthritis associated with lupus erythematosus, is now part of the therapeutic armanentarium. The ligand determines the pharmaco- and toxicokinetic profiles (e.g., only auranofin is orally available and lipophilic). All are linear and two coordinate and, unlike Au salts, are poor sources of ionic Au but tend to undergo ligand exchange reactions with protein thiols, which may underlie both their antiarthritic and cytotoxic properties.

**Figure 2:**
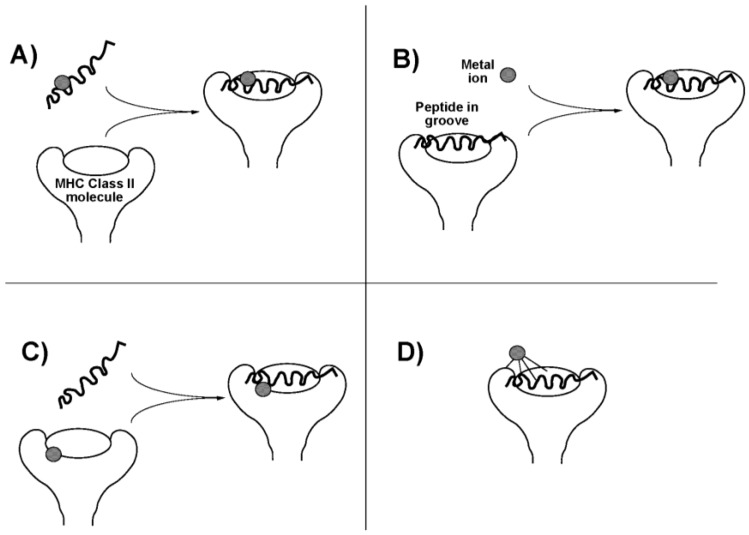
Possible scenarios for presentation of an antigenic metal hapten to; T-lymphocyte. (**A**) Metal ion binds to an antigenic peptide before association with the major histocompatability complex (MHC) molecule on the cell surface; (**B**) Antigenic peptide associates with MHC molecule before binding the metal; (**C**) Metal ion binds with the MHC molecule before recruitment of the antigenic peptide; (**D**) Metal ion binds to the intact peptide-MHC complex, perhaps requiring ligands from multiple sources. Adapted from [18]. Copyright 2004, IUPAC.

The occurrence of these ligand exchange reactions may enhance the allergenicity of the therapeutic Au compounds. Au(I) compounds induce a delayed (Type IV) hypersensitivity reaction. In this cell mediated reaction, CD4+ T-lymphocytes recognize antigen complexed with major histocompatibility complex (MHC) class II molecules on antigen-presenting cells. In the case of a metal, an antigenic peptide hapten interacts with the metal, and this may occur in any of four ways (Figure 2). The metal might bind to the antigenic peptide either (i) before or (ii) after it associates with the MHC molecule, in either case changing the conformation or presentation of the peptide. It might also (iii) interact with the MHC molecule itself on the antigen-presenting cell, before peptide binding occurs, to influence subsequent peptide binding and presentation, or it might (iv) interact with the MHC protein component only in the intact complex (perhaps requiring ligands from both peptide and MHC protein), again changing the way the complex is seen by the T-cell receptor [18]. These mechanisms may depend on speciation of the Au atom. Pretreatment of antigen-presenting cells with either the Au(I) compounds aurothioglucose or aurothiomalate, or with the Au(III) salt HAuCl_4_, or with HAuCl_4_ treated with glutathione to reduce the metal to Au^+^, all; inhibited T-lymphocyte proliferation, suggesting direct binding to the MHC molecule (mechanism (iii)) [19,20]. On the other hand, disodium Au(I)thiomalate also forms complexes with MHC-binding peptides containing two or more cysteine residues and inhibits T-cell receptor binding (mechanism (i) or (ii)) [21].

The mechanisms of action of the gold compounds in arthritis has never been fully elucidated, and side effects such as manifestations of immunosensitization and nephrotoxicity (including immune-complex glomerulonephritis) have lead to decreased use of chrysotherapy over the past few years. However, other therapeutic uses of Au are being explored. Au(III) is a strong oxidizing agent of generally higher toxicity than Au(I), and both the Au(III) and the Au(I) antiarthritic drugs are reduced to Au(0) by biological reductants such as thiols. This increased reactivity, as well as the fact that Au(III) is isoelectronic with Pt(II) and forms square planar complexes like the Pt complexes discussed below, has lead to examination of the antitumour activity of Au(III) complexes [17], notably of the pentacoordinate 2-[(dimethylamino)methyl]phenyl group (damp), as in (damp)AuCl_2_ (Figure 1). Coming full circle, Eiter *et al.* [22] have more recently shown that cationic Au(I) complexes; derived from a class of analogous Pt(II) antitumor agents containing a 1-[2-(acridin-9-ylamino)ethyl]-1,3-dimethylthiourea ligand show poor antitumor activity but strong, selective antimicrobial activity against *Mycobacterium tuberculosis*.

### 4.2. Platinum Compounds

**Figure 3:**
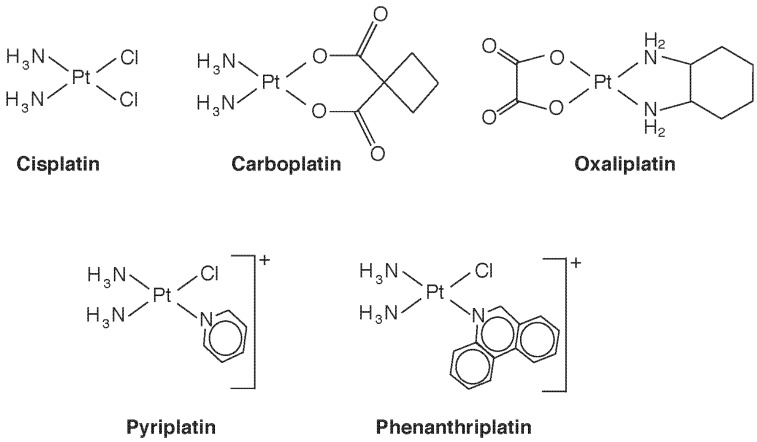
Some therapeutic platinum(II) species discussed in the text.

The Pt(II) compound *cis*-diamminedichloroplatinum (Figure 3), which goes by its generic drug name cisplatin, was the first Pt compound used as an anti-cancer agent. Now widely used against a varety of cancers, it is nearly curative in early testicular cancer. It binds to DNA, causing cross-linking, leading the cell to sense DNA damage and attempt repair [23]. When repair is unsuccessful, cell death by the intrinsic pathway of apoptosis is triggered [24], both through p53-dependent and -independent mechanisms. The initial step of binding results when Cl^−^ is displaced and the Pt center becomes available to bind to a purine base, often guanidine. Displacement of the second Cl^-^, often by a second purine, results in cross-linking. The stereochemistry is important, the *trans* isomer being all but inactive pharmacologically. The requirement for *cis*-chloride ligands in square planar geometry was thought to be necessary for formation of the major intrastrand crosslink, which involves adjacent guanosine residues, but active compounds with *trans* sterochemistry, Pt(IV) complexes, monofunctional Pt(II) complexes, and polynuclear Pt compounds have all now been identified with antitumour activity [23]. Conformational changes induced in Pt-bound DNA also inhibit transcription by inhibiting DNA binding of a number of proteins, including transcription factors, DNA repair enzymes, and RNA polymerases. Thus, multiple mechanisms of action are at play, and a study of structure-activity relationships is providing potentially new Pt species that circumvent drug resistance by targeting specific mechanisms. One example is the monofunctional *cis*-[Pt(NH_3_)_2_(pyridine)Cl]^+^ (pyriplatin, Figure 3), which induces only a small conformational change in DNA upon binding. In contrast to ciplatin, which inhibits procession of RNA polymerase at the translocation step, the pyriplatin DNA adduct arrests RNA strand growth at a post-translocation event [25], and imparts a different spectrum of tumour activity. Recognizing that steric hindrance played a role in pyriplatin activity, its pyridine ligand was replaced with phenanthridine to produce phenanthriplatin (Figure 3), which, like monofunctional pyriplatin, is also expected to have unique properties of cellular uptake and inhibition of transcription [25]. Binding of Pt complexes to proteins, such as cysteine proteases, copper proteins, and zinc-finger proteins, is another developing area, that will surely lead to a new outlook on species-dependent effects on Pt cytotoxicity and anticancer potential [26]. Recent reviews on Pt complexes that additionally consider Ru, Rh, Pd, Os, Ir, and Au also include interesting historical aspects [27,28].

Platinum, like Au, is capable of causing immunosensitization. Workers in Pt refineries and engaged in the manufacture of Pt-based catalysts develop an immunoglobulin E-mediated immediate (Type I) hypersensitivity, distinct from the Type IV T-cell-mediated reaction discussed above for Au. The species dependence is quite unusual, and no doubt involves protein binding, rather than DNA adduct formation. Sensitization is apparently restricted to halogenated Pt salts such as (NH_4_)_2_[Pt(IV)Cl_6_] where the halogen is a good leaving group [2]. When the halide is not a ligand coordinated to Pt, but is present as an ion, as in [(NH_3_)_4_Pt(II)]Cl_2_, sensitization does not occur despite the greater water solubility of the latter compound. Nor are neutral Pt compounds immunosensitizers. It is likely that the halogen leaving group increases the reactivity of the Pt center to functional groups such as R–S–CH3, R–SH, and imidazole in proteins. The Pt-based chemotherapeutic drugs cisplatin, carboplatin, and oxaliplatin also cause type I hypersensitivity reactions, but the mechanisms appear to be more complex: cross reactivity does not occur, allowing substitution of one drug with another when sensitivity arises [2].

## 5. Chelation Therapy as an Exercise in Speciation: Fe and U

### 5.1. Chelation for Iron Overload

The low aqueous solubility of Fe^3+^ has determined the evolution of very specific pathways for Fe accumulation by organisms, and Fe deficiency remains a major concern worldwide, particularly for maternal and child health. Nevertheless, several genetic diseases cause primary hemochromatosis (reviewed in [29]), an excessive accumulation of Fe that can be treated with periodic phlebotomy. On the other hand, various anemias, and particularly β-thalassemia major, which interferes with hemoglobin synthesis, lead to anemias that must be treated with repetitive blood transfusions and these cause a secondary Fe overload (“secondary hemochromatosis”) that necessitates chelation to remove the excess Fe. The design of effective chelation therapy for secondary Fe overload has been a *tour de force* of speciation analysis, both in terms of understanding the natural speciation of excess Fe in the body, and then in predicting the toxicokinetics of therapeutically-derived Fe chelates.

In the healthy adult human, most of the body’s 3–4 g of Fe is bound to hemoglobin in circulating erythrocytes, and to a lesser extent to myoglobin in muscles, with a small amount bound to transferrin in the plasma, and trace amounts occurring in various metalloenzymes. About 25% is stored in the liver. When excessive amounts of iron build up in hemochromatotic states, the Fe-binding sites in plasma transferrin become saturated, and excess plasma Fe is referred to as non-transferrin-bound iron (NTBI), consisting of low molecular weight complexes, predominantly of Fe(III)citrate. This NTBI is taken up by soft tissues and their capacity for ferritin synthesis is upregulated. When the capacity for ferritin storage is itself overcome, excess Fe is precipitated in a form called hemosiderin, probably consisting mainly of precipitates and insoluble protein complexes of Fe as the hydroxide Fe(OH)_3_ and oxihydroxide FeO(OH). Thus, whereas in healthy liver most Fe is stored bound to ferritin, with trace amounts found in internalized transferrin and hemoproteins, we were able to fractionate liver biopsy samples from patients receiving chelation therapy for; excessive Fe overload and found the Fe content to be transferrin (<1%), hemoproteins (4%), ferritin (22%) and hemosiderin (74%) [30]. A similar profile is found in other organs such as heart, although they account in absolute terms for much lower amounts of body Fe stores. Liver damage and cardiotoxicity are the most important cuases of death in Fe overload. Targeting excess Fe for chelation is, then, an exercise in understanding the speciation of the Fe stores. While Fe(III) bound to transferrin (logK_a_ ~ 22) is difficult to remove, NTBI should be readily accessible to chelation. In the cell, a labile Fe pool in transit from internalized transferrin to ferritin stores, or mobilized from ferritin (e.g., for use in cytochrome or Fe-sulfur cluster synthesis), should be accessible, whereas hemosiderin deposits are expected to be quite inert. The redox chemistry of Fe becomes important here, too. Release of Fe from ferritin may involve reduction of Fe(III). Unlike Fe^3+^, which begins to hydrolyze at pH 1, Fe^2+^ is relatively free from hydrolysis at pH 7 [31], making ferrous ion available for diffusion and transport. However, the fact that long-term chelation therapy is able to normalize body Fe stores and distribution indicates that each of these Fe pools is amenable to chelation, either directly with a range of kinetic accessibilities, or indirectly following redistribution of equilibria after the more labile species are first depleted.

**Figure 4:**
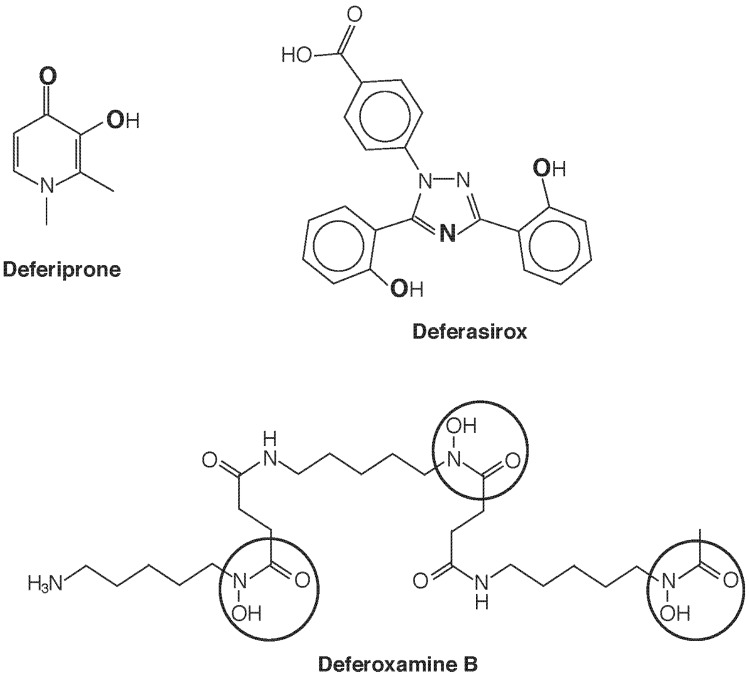
Iron chelating agents in clinical use. Deferiprone chelates Fe^3+^ with 3:1 stoichiometry in a bidentate fashion upon deprotonation of the hydroxyl group, using the two O atoms shown in bold. Deferasirox is a tridentate Fe^3+^ chelator that, upon deprotonation of the two phenolic groups, uses the three bold atoms (two O and one N) to bind in a 2:1 complex. Hexadentate deferoxamine B forms a 1:1 complex with Fe^3+^ using the six O atoms of the three circled hydroxamate groups.

Introduction of a chelating agent adds another dimension of speciation, as now the species of chelator-bound Fe will determine mobilization, redistribution, and excretion. Currently three chelating drugs are approved for use in most countries deferoxamine (DFO), deferiprone (dfp), and deferasirox (Figure 4). One notable difference in these three agents is their denticity. Deferoxamine is hexadentate and satisfies the six sites for octahedral coordination of Fe(III) in 1:1 stoichiometry. Deferasirox is tridentate and forms 2:1 complexes with Fe(III), while deferiprone is bidentate and requires 3:1 stoichiometry for full coordination. A consequence is that with the latter two structures, equilibria can be anticipated [e.g., dfp_3_Fe ⇌ dfp_2_Fe(H_2_O)_2_^+^ ⇌ dfpFe(H_2_O)_4_^2+^] that leave the metal center accessible and potentially active as a Fenton catalyst. Such considerations seem to be of less concern than once feared, as a wealth of clinical experience has proven the safety of these drugs, and there use in combination has been the subject of several successful clinical trials. The greatest impact of speciation seems to be in determining cellular and subcellular access and elimination.

Because of their physicochemical characteristics, DFO enters cells poorly whereas defriprone and deferasirox cross membranes more readily. Thus, DFO accesses mainly NTBI and the resultant ferrioxamine complex is excreted in the urine, whereas deferiprone and deferasirox remove cellular Fe [32]. An exception is the hepatocyte that has a facilitative mechanism for DFO uptake, so DFO also accesses intracellular ferritin in the liver, destabilizing it for degradation by lysosomes [33], and the liberated Fe(III) is bound and excreted in the bile [32]. Deferiprone and deferasirox both chelate cytosolic labile Fe, but their Fe(III) complexes undergo renal and heptobiliary excretion, respectively. Whereas DFO is effective in removing Fe from both liver and heart, deferasirox is more effective in eliminating hepatic Fe and deferiprone is more effective in removing cardiac than hepatic Fe [34]. Deferiprone facilitates transfer of extracellular Fe into mitochondria and nuclei, but also from intracellular compartments to extracellular apotransferrin and between endosomes and nuclei [32]. The access of deferiprone to mitochondrial Fe may offer an advantage in treating the complications of cardiac and endocrine Fe overload. While these differential effects suggest the possibility of combination therapy, combinations of deferiprone or deferasirox with DFO have not yielded any conclusive results by meta-analysis that support a particular regimen [35].

These considerations also become important as Fe chelation therapy is used to target additional disease processes. Friedreich ataxia is now known to be due to a mutation in the gene coding the mitochondrial protein frataxin, impairing utilization of Fe for iron-sulfur cluster biosynthesis and resulting in accumulation of toxic amounts of Fe in the mitochondrion. Deferiprone, which together with its Fe(III) chelate crosses membranes readily, has been used in clinical trials to treat Friedreich ataxia, hoping to exploit both its ability to cross the blood-brain barrier and to access intracellular compartments. In one such trial, improvement in disease progression was shown in patients with less severe disease, although administering higher levels of chelator worsened ataxia [36], possibly due to excessive mobilization of Fe as an available species. Myelodisplastic syndromes characterized by ineffective erythropoiesis and anemia lead to secondary Fe overload when patients become dependent on chronic blood transfusion. All three chelators have been tried in treating these patients, and protocols are governed mainly by side effects [37].

### 5.2. Chelation for Uranium Poisoning

The once little-studied field of U poisoning and its treatment gained prominence with the introduction of depleted uranium (DU) weaponry, particularly by the U.S. military in the Persian Gulf war. Briner has reviewed the toxicity of DU [38], and Katz in this series has described the occurrence of DU in the environment, and reviewed the toxicokinetics of the products that arise from its military use [39]. The toxicity of products derived from DU is a result both of radiotoxicity (the isotopes in naturally-occurring uranium—^238^U, ^235^U, and ^234^U—all being α-particle emitters) and chemical toxicity of metallic species, with the latter apparently dominating in human exposures. For instance, uranyl salts are capable of inhibiting a number of enzymes [39]. Thus, the chemical speciation of U becomes an important determinant of the pattern of toxicity, and of the effectiveness of chelation treatment.

DU refers to the nearly pure non-fissionable ^238^U left behind when U is enriched in fissionable ^235^U. This DU “waste”, when fabricated into projectiles, becomes a “kinetic penetrator” capable of piercing armoured tanks and concrete bunkers. In doing so, it burns to uranium(IV)oxide vapour, consisting of UO_2_ particles of varying sizes. Especially in the fraction with particle sizes less than about 0.5 µm [40], UO_2_ dissolves in water and is readily oxidized to the U(VI) uranyl ion, UO_2_^2+^. Thus, once absorbed into the blood stream, the oxide exists predominantly as uranyl ion partitioned between transferrin and small molecules such as citrate and bicarbonate. The association constant of UO_2_^2+^ with apotransferrin is about four orders of magnitude less than that of Fe(III) [40]. Larger particles are retained with a long biological half-life; smaller particles are soluble and produce toxic symptoms other than acute radiotoxicity, notably renal failure, as well as bone abnormalities and toxicity to other organs.

The current state of chelation therapy for U intoxication suggests the efficacy of citrate [40]. Sodium citrate reduced the lethality of uranyl nitrate, UO_2_(NO_3_)_2_, administered to dogs, and it joins a number of other chelators shown to be effective in mice. While ethylenediaminetetraacetic acid (EDTA) is effective in increasing urinary excretion of UO_2_^2+^ in mice, it did not achieve a significant degree of mobilization of U from bone. Other divalent ion chelators such as diethylenetriaminepentaacetic acid (DPTA) and ethylene glycol tetraacetic acid (EGTA) are also protective, as are the catechol-based Fe(III) chelators 4,5-dihydroxy-1,3-benzenedisulfonate (Tiron) and gallic acid. In fact, of sixteen agents tested, Tiron showed the greatest protection against acute UO_2_^2+^ toxicity [41], and the related catechol, catechol-3,6-bis(methyleiminodiacetic acid) (CBMIDA), has also been shown to increase urinary excretion of U in rats given DU by intraperitoneal or intramuscular injection (see [38]). Although these latter routes of administration are of limited relevance to most human exposures, which are from inhalation of particulates, the observation supports the effectiveness of Fe(III) chelators in removing UO_2_^2+^ salts from the body. Desferrioxamine forms a stable complex with UO_2_^2+^ at physiological pH, and may be of use in a clinical setting, but citrate and bicarbonate both displace UO_2_^2+^ from DFO, suggesting that citrate may be an agent of choice in prophylaxis and treatment of UO_2_^2+^ toxicity, and to this end ingestion of citrate-rich foods such as fruit juice may be recommended in those potentially exposed to UO_2_-containing particles [40]. However, it is interesting to note that co-administration of D-fructose intraperitoneally with subcutaneous UO_2_^2+^ caused a marked (ca. seven-fold) decrease in the LD_50_, and so the ingestion of beverages with a high content of sucrose or fructose should be viewed with caution in a scenario of DU artillery deployment. This is an interesting example of studies on chelation therapy for metal toxicity suggesting a dietary modification based on a supposed change in speciation.

## 6. Conclusions

The biological effects of metals and semi-metals are dominated by their speciation. Imprecise terms like “heavy metal” or “toxic metal” should be avoided, and generalizations about an element that do not refer to specific chemical species can cause confusion. Elemental speciation is manifest at various structural levels, and among them electronic structure (oxidation state), the nature of organic and inorganic bonds and ligands, and macromolecular complexes are important in determining biological behaviour. When speciation is taken into account, our understanding of mechanisms of toxicity, of how to remove harmful metals from the body, and of how to exploit metal compounds as drugs are all enhanced; without these considerations, misleading generalities often occur.
